# Microemulsion of Cinnamon Essential Oil Formulated with Tea Polyphenols, Gallic Acid, and Tween 80: Antimicrobial Properties, Stability and Mechanism of Action

**DOI:** 10.3390/microorganisms11010002

**Published:** 2022-12-20

**Authors:** Wei Wang, Yin-Feng Chen, Ze-Feng Wei, Jing-Jing Jiang, Jia-Qian Peng, Qi-Tong He, Wen-Ying Xu, Hui-Min Liu

**Affiliations:** 1 School of Perfume & Aroma and Cosmetics, Shanghai Institute of Technology, Shanghai 201418, China; 2 Engineering Research Center of Perfume & Aroma and Cosmetics, Ministry of Education, Shanghai 201418, China

**Keywords:** cinnamon essential oil, tea polyphenols, gallic acid, microemulsion, antimicrobial activity, antioxidant activity, stability, irritant, antimicrobial mechanism

## Abstract

The objective of this article was to combine tea polyphenols, gallic acid, and cinnamon essential oil to construct a natural extract-complex microemulsion system (NMs) with good antibacterial activity, antioxidant activity, and stability, as well as low irritation. NMs were characterized by particle size distribution, electrical conductivity, and light transmittance. The stability, as well as the antimicrobial, antioxidant, irritation, and antimicrobial mechanisms, of NMs were also studied. The results showed that NMs had a significant antimicrobial function against *Staphylococcus aureus*, *Escherichia coli*, *Pseudomonas aeruginosa*, *Candida albicans* and *Aspergillus brasiliensis*. The minimum inhibitory concentrations were 156 μg/mL, 62.5 μg/mL, 125 μg/mL, 250 μg/mL, and 125 μg/mL, respectively. Through the cell membrane permeability test and growth curve test of bacteria and fungi, we concluded that the NMs’ mechanism of action on bacteria and fungi could be interpreted as NMs mainly altering the permeability of cell membranes to inhibit the growth of bacteria and fungi. The results of this study have important implications for utilizing plant extracts as natural preservatives for food and cosmetics.

## 1. Introduction

Many compounds from natural sources have been reported to have medical, cosmetic, and pharmaceutical applications [1]. Essential oils (EOs) are metabolic products of secondary substances in aromatic plants, and have recently garnered plentiful attention because of their antibiofilm and antibacterial activities [2]. EOs originate from aromatic plants or parts of aromatic plants and include volatile components, primarily terpenes, terpenoids, and phenylpropenes [3]. With the status of being generally recognized as safe, the application of EOs in food preservation has become an area of focus [4]. Cinnamon essential oil (CEO) has been used as flavoring agent, fragrance agent, and antimicrobial agent in foodstuffs and cosmetics [5]. Combining several EOs or EOs with different antimicrobial properties may allow for synergistic antibacterial activity, effectively decreasing the required usage of EOs or antimicrobials [6,7,8,9]. Besides antibacterial properties, many EOs have been reported to have antioxidant, antitumor, antifungal, and anti-inflammatory properties [10]. Despite their well-documented characteristics, the application of EOs is limited in food and cosmetics due to their low water solubility, high volatility, pungent flavor, and instability to special conditions such as light, temperature, and humidity.

Oil-in-water (O/W) emulsions, nanoemulsions, and microemulsions are commonly prepared to disperse EOs in water systems [4]. Microemulsions are homogeneous dispersion systems composed of a water phase, oil phase, surfactant, and cosurfactants, and exhibit thermodynamic stability, transparency, or clarity [11,12]. Microemulsions are mainly divided into three types: water-in-oil (W/O), O/W, and discontinuous [13]. In the fields of materials, cosmetics, pharmaceuticals, food, and other industries, microemulsion technology has developed rapidly [14,15,16]. For example, carvacrol microemulsion can enhance transdermal absorption and anti-inflammatory activity [17]; pharmaceuticals and cosmetics can be made more effective by using microemulsion systems designed for optimal permeability [18].

Phenolic compounds combined with various proteins have been proven to improve antioxidant capability and stabilize the emulsion interface structure [19,20]. Gallic acid (GA) is a typical phenolic compound widely used in the fields of food and medicine because of its antioxidant and antibacterial properties [21,22,23]. Tea polyphenols (TP) are polyphenolic mixtures distilled from green tea [24,25], with higher antioxidant potential than traditional synthetic antioxidants (butylated hydroxy anisole, butylated hydroxytoluene, and tertbutyl hydroquinone), as well as a natural antioxidant, vitamin E [26,27]. The addition of natural polyphenolic compounds to the microemulsion is beneficial for improving the stability of the microemulsion. Jiang et al. showed that GA can improve the emulsion stability and thermal stability of microemulsions [21]. In addition, Lan et al. showed that TP can improve the stability of microemulsions [28].

With the advancement of scientific technology, society’s research on preservatives has become more in-depth, and many traditionally used preservatives have been confirmed to have specific adverse effects. For example, propylparaben (PP), which is commonly applied in the fields of food and cosmetics, is irritating to the eyes, respiratory system, and skin. It is an inevitable new trend to develop mild, low-toxicity, efficient, and environmentally friendly natural preservatives to replace, in whole or in part, traditional chemical preservatives. By adequately combining different plant extracts, excellent and broad-spectrum antimicrobial activity can be obtained. Therefore, it has become a feasible scheme to construct a safe and effective antiseptic system through the combination of natural antiseptic active components. In this paper, a microemulsion system with bacteriostatic effect was prepared by taking CEO, TP, and GA from natural sources as active antibacterial components by microemulsion technology. The present work aims to (i) construct a stable microemulsion system composed of different active ingredients, (ii) determine the in vitro inhibitory activity of the microemulsion against *Staphylococcus aureus* (*S. aureus*), *Escherichia coli* (*E. coli*), *Pseudomonas aeruginosa* (*P. aeruginosa*), *Candida albicans* (*C. albicans*), and *Aspergillus brasiliensis* (*A. brasiliensis*), (iii) evaluate antioxidant properties and stability of, and irritation caused by, the microemulsion, and (iv) explore the antibacterial mechanism of the microemulsion. This study was carried out to find a potential alternative to traditional preservatives with a broader antibacterial spectrum, more stable efficacy, and more excellent safety.

## 2. Materials and Methods

### 2.1. Materials

TP (99%), GA (98%), glycerol, Tween 80, ethanol, PP, isopropyl palmitate, vitamin E, and bovine albumin were bought from Macklin Biochemical (Shanghai, China). CEO (85%) was purchased from Shanghai Titan Scientific (Shanghai, China). Soybean casein agar medium (TSA), potato dextrose agar (PDA), Luria–Bertani broth (LB), and potato liquid medium (PLM) were procured from Qingdao Hope Bio-Technology (Qingdao, China). Eggs were purchased from Zhejiang Lihua Agricultural Technology Co., Ltd (Zhejiang, China).

### 2.2. Establishment of NMs

Water dilution was used to construct pseudo-ternary phase diagrams [29]. The CEO, TP, and GA were mixed, and the microphase domain of the NMs was observed by drawing a pseudo-ternary phase diagram. The diameter of the inhibition zone and the area of the microemulsion area were used as indicators to determine the proportion of active ingredients in the system. The effect of the added amount of TP in the water phase on the NMs is shown in Supplementary Figure S1, Supplementary Tables S1 and S2; the final selection of the consistency of TP in the water phase was 50 mg/mL. The effect of different concentrations of GA on the NMs is shown in Supplementary Figure S2; the final concentration of GA in glycerol was 100 mg/mL. The effect of the ratio of CEO to co-emulsifier on NMs is shown in Supplementary Figure S3 and Supplementary Table S3; the ratio of CEO to the glycerin phase was 1:1. It was finally determined that the proportion of CEO, glycerol phase, Tween 80, and water phase in the NMs of the natural extract complex microemulsion system constructed in this paper were 13.5%, 13.5%, 50%, and 23%.

### 2.3. Characterization of NMs

#### 2.3.1. Measurement of Particle Size Distribution

The NMs were diluted 100-fold with deionized water. The average droplet size was measured by laser particle size analyzer using a Malvern Zetasizer Nano-ZS (ZNS3600, Malvern Instruments Ltd., Worcestershire, UK).

#### 2.3.2. Measurement of Transmittance

The transmittance of the NMs at 200–800 nm was assessed using a microplate reader (Multiskan Sky, Thermo Fisher Scientific, Shanghai, China).

#### 2.3.3. Measurement of Electrical Conductivity

The electrodes of the conductivity meter (DDS-307A, Shanghai INESA Scientific Instrument Co., Ltd., Shanghai, China) were immersed in the NMs, and the conductivity was recorded after reaching equilibrium.

#### 2.3.4. Measurement of pH

The electrode of the pH meter (PHS-3E, Shanghai INESA Scientific Instrument Co., Ltd., Shanghai, China) was dipped into the microemulsion sample, and the pH value was recorded after reaching equilibrium.

### 2.4. Antimicrobial Test

#### 2.4.1. Determination of the Inhibition Zone Diameter

The samples were tested for bacteria and fungi using the filter paper diffusion method [30,31]. A suspension of the tested bacteria or fungi (1 × 10^7^ or 1 × 10^8^ CFU/mL) was spread on the solid media plates. The paper discs (6 mm diameter) were impregnated with 200 μL of sample and placed on the agar surface. The plates inoculated with bacterial or fungi strains were incubated for 24 h or 48 h at 37 °C or 28 °C. Bacteria were cultured on TSA medium and fungi were cultured on PDA medium. The inhibition zone diameter (mm) was measured with a ruler.

#### 2.4.2. Determination of MIC

MIC is the lowest concentration of the sample which inhibits visible bacterial or fungus growth after overnight incubation. MIC of the samples were measured by the microplate method [32]. Two-fold serial dilution of samples (dissolved in Tween 80 (1% *v/v*)) were performed in culture medium. After that, 20 μL of each bacterial or fungi suspension was inoculated. Each well included 100 µL of sample diluted in culture medium, 80 µL of the growth medium, and 20 µL of cell suspension (1 × 10^6^ CFU/mL). Bacteria were cultured on LB medium and fungi were cultured on PLM medium. Culture medium and Tween 80 (1% *v/v*) were used as blank and negative controls, respectively. After culturing at 37 °C or 28 °C with constant temperature shaking for 24 h or 48 h, the absorbance of the microplate was measured by microplate reader (Multiskan Sky, Thermo Fisher Scientific, Shanghai, China) at 600 nm.

### 2.5. Antimicrobial Mechanism of NMs

#### 2.5.1. Growth Curve

Bacterial and fungal growth curves were tested according to methods reported in the literature [33,34] with some modifications. The different samples were cultured in the medium with added *P. aeruginosa* (6 × 10^4^ CFU/mL) or *C. albicans* (3 × 10^4^ CFU/mL), and the concentration of the samples was 0.5 MIC. TP, GA, and PP were dissolved in deionized water, and CEO was dispersed with Tween 80 (1% *v/v*). Deionized water and Tween 80 (1% *v/v*) were used as solvent control. PP was a positive control, and no preservative was added as the blank control.

At specific time intervals, the *P. aeruginosa* and *C. albicans* suspension containing samples from the test culture were taken and serially diluted in sterile water. All plates were then incubated for at 37 °C or 28 °C for 24 h or 48 h, and CFU were counted by plate count method.

#### 2.5.2. Cell Membrane Permeability

##### Relative Conductivity

Relative conductivity was utilized to characterize the cell membrane permeability of bacteria and fungi in accordance with the methods from Diao et al. [35] with some modifications. The *P. aeruginosa* and *C. albicans* were washed with 5% glucose until their electric conductivities were near that of 5% glucose. The different samples were cultured in the medium with added *P. aeruginosa* (1 × 10^7^ CFU/mL) or *C. albicans* (1 × 10^5^ CFU/mL), and the concentration of the samples was 0.5 MIC. After being cultured at 37 °C or 28 °C with constant temperature shaking for 4 h or 8 h, *P. aeruginosa* or *C. albicans* were centrifuged at 10,000× *g* and 4 °C for 10 min and the conductivity of the supernatant was measured and marked as L_1_. The glucose solution was measured and marked L_0_; the glucose solution without bacteria and only containing preservatives was measured and marked as L_3_. The conductivity of *P. aeruginosa* and *C. albicans* in 5% glucose treated in boiling water for 15 min were served as the control and marked as L_2_. The permeability of the intracellular membrane can be expressed by the relative conductivity C, which is calculated as follows:

(1) $$C=\frac{L_{1}-L_{3}}{L_{0}-L_{2}}\times 100\%$$

##### Protein Leakage Assay

To further explore the effect of microemulsion on intracellular protein leakage, Coomassie Brilliant Blue (CBB) was used to measure the protein content in the bacterial and fungal suspension. The calibration curve for bovine serum albumin was established based on Abs and concentration. The *P. aeruginosa* and *C. albicans* were washed twice with 0.067 mol/L phosphate buffer solution (PBS). The different samples were cultured in the medium with added *P. aeruginosa* (1 × 10^7^ CFU/mL) or *C. albicans* (1 × 10^5^ CFU/mL), and the concentration of the samples was 0.5 MIC. After culturing at 37 °C or 28 °C with constant temperature shaking for 4 h or 8 h, *P. aeruginosa* or *C. albicans* were centrifuged at 10,000× *g* and 4 °C for 10 min. The supernatant reacted with CBB solution at 25 °C for 10 min and the absorbance was measured by microplate reader (Multiskan Sky, Thermo Fisher Scientific, Shanghai, China) at 595 nm.

##### Nucleic Acid Leakage Assay

The amount of nucleic acid leakage is often characterized by the Abs at 260 nm (Abs_260_) and is used to evaluate the permeability and integrity of cell membranes. The *P. aeruginosa* and *C. albicans* were washed twice with 0.067 mol/L phosphate buffer solution (PBS). The different samples were cultured in the medium with added *P. aeruginosa* (1 × 10^7^ CFU/mL) or *C. albicans* (1 × 10^5^ CFU/mL), and the concentration of the samples was 0.5 MIC. After culturing at 37 °C or 28 °C with constant temperature shaking for 4 h or 8 h, *P. aeruginosa* or *C. albicans* were centrifuged at 10,000× *g* and 4 °C for 10 min, and the supernatant subsequently taken to measure the Abs_260_.

### 2.6. DPPH Free-Radical-Scavenging Capacity Assay

The radical 2,2-diphenyl-1-picrylhydrazyl (DPPH) scavenging activity was determined according to the method of Lin [30] with some modifications. Vitamin E was selected as a positive control. The following formula expresses the scavenging rate:

(2) $$\mathrm{DPPH}\;\mathrm{scavenging}\;\mathrm{rate}\;\left(\%\right)=\left(1-\frac{A_{1}-A_{2}}{A_{3}-A_{0}}\right)\times 100\%$$

where A_1_ is the Abs after the reaction of sample with DPPH solution, A_2_ is the Abs of sample mixed with ethanol, A_3_ is the Abs of DPPH solution mixed with ethanol and A_0_ is the Abs of the solvent (ethanol).

### 2.7. Stability Test

#### 2.7.1. Centrifugation Stability

The NMs were centrifuged at 5000× *g* (relative centrifugal force) for 30 min to assess stability. The samples were observed for instability phase separation, creaming, or flocculation [36].

#### 2.7.2. Thermal and Storage Stability

The NMs were placed at high temperature (45 °C), low temperature (−5 °C), and normal temperature (20 °C) for 15 d, 30 d, 60 d, and 90 d, then centrifuged at 10,000× *g* and 4 °C for 10 min for observation. The stability of NMs was characterized by the change in inhibition zone, and the rate of decrease in the diameter of the inhibition zone was calculated after 90d; the calculation formula is as follows:

(3) $$\mathrm{Rate}\;\mathrm{of}\;\mathrm{decline}\;\left(\%\right)=\frac{d_{0}-d_{90}}{d_{0}}\times 100\%$$

where d_0_ is the inhibition zone of the NMs on the 0th day, and d_90_ is the inhibition zone of the NMs on the 90th day.

### 2.8. Irritation Assay

NMs were investigated for irritation using the hen’s egg test on the chorioallantoic membrane (HET-CAM), which was a prospective method for evaluating the irritation potential of samples by observing adverse changes in the chorioallantoic membrane of the hen’s egg after exposure to the test chemicals [17,37]. HET-CAM tests were performed according to the method provided in the literature [38]. The chorioallantoic membrane (CAM) was exposed to 300 μL of one of each of the following substances: (1) 0.9 % *w/v* sodium chloride solution (negative control), (2) 0.1 mol/L NaOH solution (positive control), (3) deionized water and isopropyl palmitate (solvent control), (4) PP diluted to 0.05%–0.3% with isopropyl palmitate (benchmark control), and (5) NMs diluted to 0.05%–0.3% with deionized water.

### 2.9. Statistical Analysis

Data was expressed as mean ± standard deviation (*n* = 3). One-way analysis of variance and Duncan’s multiple range tests were carried out to determine significant differences (*p* < 0.05) between the means by SPSS 26 v.26 (International Business Machines Corporation, Armonk, NY, USA) and Origin 2022 v.9.9 (OriginLab Corporation, Northampton, MA, USA).

## 3. Results and Discussion

### 3.1. Characterization of Microemulsions

**Figure 1:**
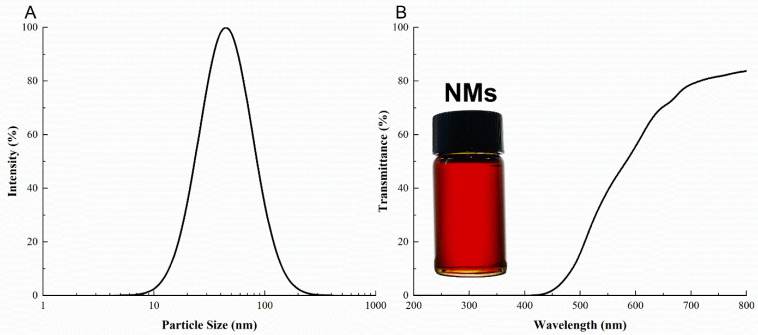
Particle size and light transmittance of NMs. (**A**) particle size distribution, (**B**) light transmittance. Each data represents means ± SD (*n* = 5).

At 25 °C, the pH of NMs was 5.57 ± 0.03, and the conductivity was 32.4 ± 0.2 μS/cm. The pH of the NMs was found to be compatible with the skin, and conductivity data indicated that the NMs were O/W microemulsions. In addition, no phase separation was observed in the dilution of NMs with deionized water. As shown in Figure 1A, the average particle size of the NMs was 44.51 ± 0.83 nm, which was less than 100 nm and under the microemulsion category. The NM was optically homogeneous, transparent, and slightly brown. Figure 1B shows that NMs have good light-shielding properties in the ultraviolet region and good light transmittance in the visible light area. The transmittance reaches 70.1% at 650 nm, consistent with the generally good transmittance of microemulsions. Regardless, the NM was not hierarchical after 5000× *g* centrifugation for 30 min, and thus, could be identified as a microemulsion.

### 3.2. Analysis of Antimicrobial Properties of NMs

Table 1 shows the MIC values of the bacteriostatic active ingredients TP, GA, CEO, and NMs, with PP as a positive control. The MICs of NMs against *S. aureus*, *E. coli*, *P. aeruginosa*, *C. albicans*, and *A. brasiliensis* were 156 μg/mL, 62.5 μg/mL, 125 μg/mL, 250 μg/mL, and 125 μg/mL, respectively. This result is close to the MIC value of PP, indicating that NMs have the potential to replace PP. Notably, the inhibitory effect of NMs on *C. albicans* and *A. brasiliensis* was remarkable, apparently related to the excellent antifungal ability of CEO. The CEO has a strong antibacterial ability. Still, its hydrophobicity, behavior as a strong irritant, spicy flavor, and other properties make it difficult to directly add CEO as a raw material to cosmetic products.

### 3.3. Antioxidant Capacity Assay

**Figure 2:**
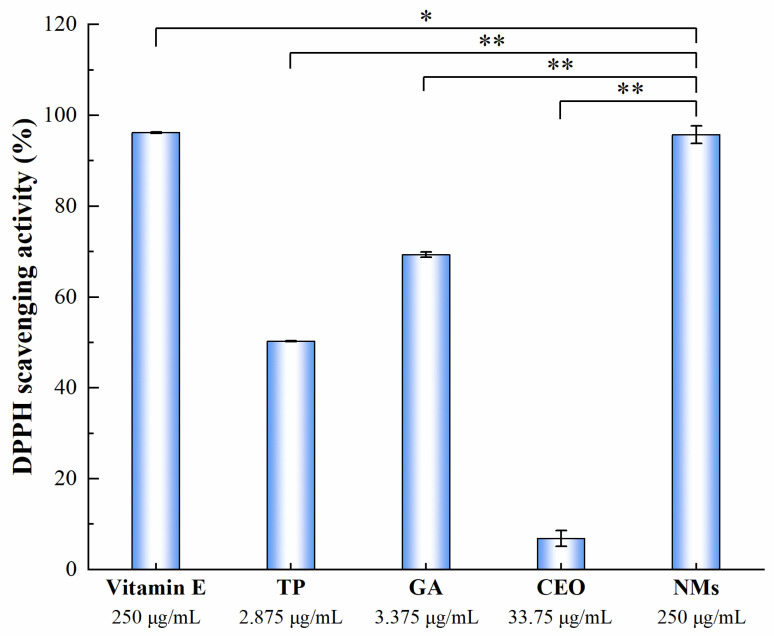
DPPH free-radical-scavenging capacity of NMs. Each value represents means ± SD (*n* = 3). * and ** indicate statistical difference at *p* < 0.05 and *p* < 0.01 levels.

Free-radical-scavenging activity is considered to be one of the primary mechanisms exhibited by antioxidants to delay oxidative processes [32]. According to the data in Table 1, when the MIC of NMs was 250 μg/mL, it had broad-spectrum antimicrobial capacity. Therefore, it was reasonable to select this concentration for antioxidant testing. The antioxidant effect of NMs was shown in Figure 2. Compared with the control group, the DPPH scavenging activity of NMs was significantly different (*p* < 0.05). At the concentration of 250 μg/mL, the DPPH radical-scavenging activity of NMs was comparable to that of vitamin E. Compared with TP, GA, and CEO, the scavenging activities were improved by 45.45%, 26.42%, and 88.88% for DPPH with significant differences (*p* < 0.01). This meant that the potent antioxidant capacity of NMs was mainly related to TP and GA. Jiang et al. reported that PV–GA complexes could significantly increase the antioxidant capacity of PV–GA/CLA microemulsions [21]. Therefore, the enhancement of free-radical-scavenging activity in our NMs could be mainly due to the combined antioxidant effect of TP and GA. The smaller particle size and the increase in chemical reaction interface could partly contribute to the improved free-scavenging activity of NMs [19].

### 3.4. NMs Stability Analysis

The stability of microemulsions is usually characterized by high- and low-temperature storage, centrifugation, freeze–thaw cycling, and thermal–cold cycling. After centrifugation at 5000× *g* for 30 min, NMs maintained the same clear and transparent appearance without phase separation. This demonstrated that a layer of surfactant and cosurfactant is strong enough to protect the oil droplets from phase separation due to centrifugal force, and that the formulation is very stable [39].

**Figure 3:**
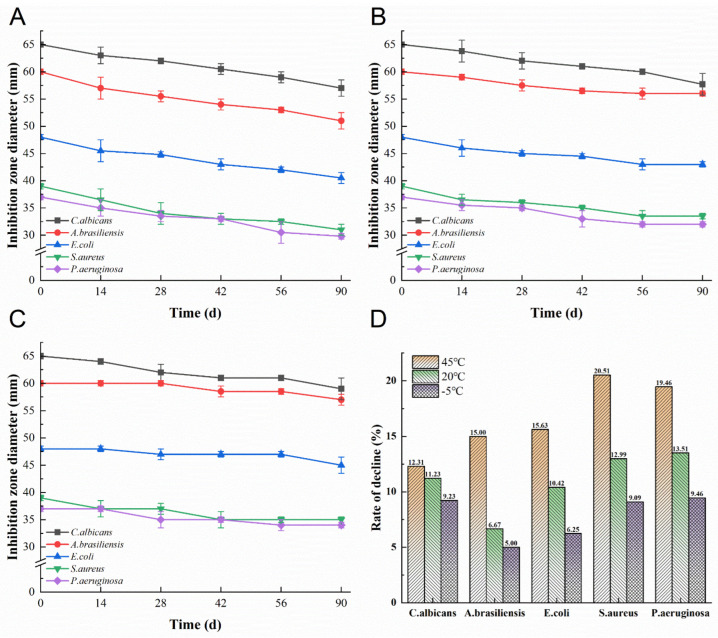
The effect of NMs on antibacterial activity at different temperatures. (**A**) 45 °C, (**B**) 20 °C, (**C**) −5 °C, (**D**) rate of decline. Data obtained from triplicate experiments are expressed as mean ± SD (*n* = 3).

Rate of decline is strong enough to protect the oil droplets from phase separation due to centrifugal force, and the formulation is very stable [39]. The NMs were stored at 45 °C, 20 °C, and −5 °C for a three-month bacteriostatic stability study. At the end of the storage test, the microemulsions at different temperatures still had good light transmittance. The color of NMs stored at high temperature has obvious signs of deepening, the color of NMs stored at room temperature is slightly darkened, and the color of NMs stored at low temperature has no obvious change. The oxidation of TP may cause the color change in NMs during storage. Figure 3 shows the diameter changes of the inhibition zone of NMs at 45 °C, 20 °C, and −5 °C for 0–90 days. It can be seen from Figure 3 that low temperature (−5 °C) has a minor effect on the antibacterial activity of NMs, and high temperature (45 °C) has the most significant effect on the antibacterial activity of NMs. Furthermore, the NMs under the three temperature conditions were centrifuged at 10,000× *g*, 4 °C, and 10 min without delamination. Although the inhibition zone diameter reduction rate changed significantly with the temperature at the end of the storage test, especially for *S. aureus* and *P. aeruginosa*, the NMs still had good bacteriostatic activity. Notably, the bacteriostatic activity of NMs against *C. albicans* was minimally affected by temperature. This may be because the oxidative decomposition of the bacteriostatic active components (CEO, TP, and GA) in NMs is promoted under the condition of higher temperature, resulting in NMs’ decreased inhibition of *S. aureus*, *E. coli*, *P. aeruginosa*, *C. albicans,* and *A. brasiliensis*. In addition, NMs are O/W microemulsions, and the TP in the water phase and the GA in the glycerol phase will be oxidized first, which will protect the CEO from oxidation. According to Table 1, both TP and GA have inhibitory effects on *S. aureus*, *E. coli*, and *P. aeruginosa*. With the oxidation of TP and GA, the antibacterial ability of NMs against *P. aeruginosa* and *S. aureus* was significantly reduced. This result is in line with a study which observed that the addition of TP can effectively retard oxidation in the three edible oils (corn, soybean, and sunflower) [28]. In addition, adding antioxidants to the emulsion can improve its storage stability of the emulsion. For example, Yang et al. reported that the addition of appropriate antioxidants (β-carotene, tanshinone, and black tea extract) into the citral-encapsulated emulsion could significantly improve the antioxidant properties, resulting in NMs with good stability [13]. Briefly, this study simulated changes in the stability of microemulsions in the refrigerator, at room temperature, and in high-temperature storage or transportation conditions by testing metrics at −5 °C, 20 °C, and 45 °C, which are significant temperatures in the food, pharmaceutical, and cosmetic fields.

### 3.5. NM Irritation Analysis

**Figure 4:**
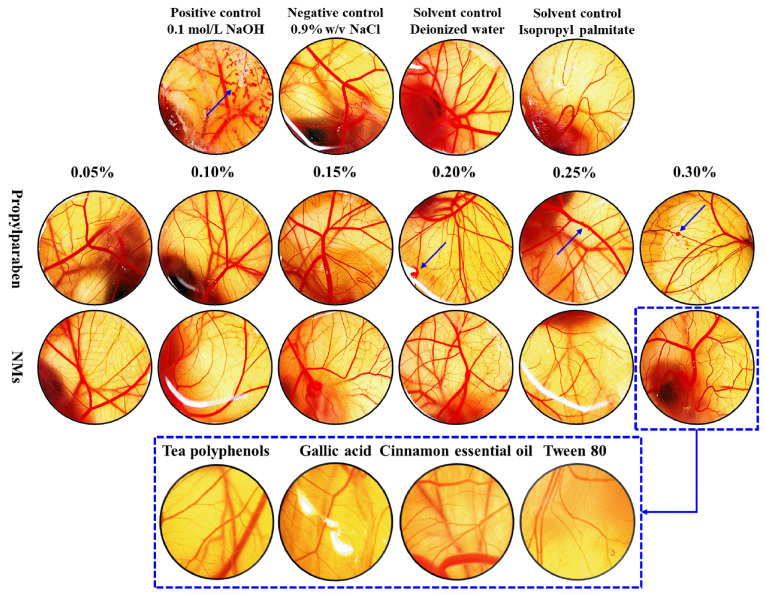
The results of HET-CAM. Effect of positive control (0.1 mol/L NaOH), negative control (0.9% *w/v* NaCl), solvent control (deionized and isopropyl palmitate), PP solution, and NMs, which were composed of deionized water and isopropyl palmitate, on the chorioallantoic membrane after 5 min. Blue arrows indicate positive control.

The irritation of skin caused by microemulsions is a critical issue, and the irritation potential of microemulsions were determined using the HET-CAM assay [17]. Compared with the positive control, where lysis of blood vessels occurred within 30 s, 0.9% (*w/v*) NaCl, deionized water, and isopropyl palmitate were found to be non-irritant. The comparative analysis of Figure 4 showed that PP was mildly irritating when its concentration was 0.2%–0.3% and the NMs was non-irritating when the concentration was 0.05%–0.3%. Based on the safety assessment result of PP and considering the concerns related to potential endocrine-disrupting properties, the Scientific Committee on Consumer Safety has concluded that PP is safe for use as a preservative in cosmetic products up to a maximum dosage of 0.14% [40]. A concentration of 0.3% of NMs was the efficient antibacterial concentration determined by the challenge of the anti-corrosion experiment. In addition, when the concentration of NMs was 0.3%, the NMs’ components, including TP, GA, CEO and Tween 80, were shown to be non-irritant. The results indicated that the formulation of antibacterial microemulsion studied was safe and effective. It has been reported that both microemulsions and essential oil solutions may induce irritation [17]. Therefore, it is necessary to study the safety of the formulation of essential oil microemulsions.

### 3.6. Antimicrobial Mechanism Analysis of NMs

Although some microemulsions and nanoemulsions containing CEO have been prepared by different methods and their antibacterial activities and stability studied, the antibacterial mechanisms of microemulsions and nanoemulsions have not been studied in detail [36,41,42,43,44]. It can be seen from Table 1 that TP and GA have potent inhibitory effects on bacteria and weaker inhibitory effects on fungi. In contrast, CEO has a better antifungal effect. This paper studied the NMs’ antibacterial mechanism with *P. aeruginosa* and *C. albicans*. The currently recognized antibacterial mechanisms may be increased cell membrane permeability, enzyme activity inhibition, and genetic material destruction or inactivation [45,46]. Based on the preliminary research, the antimicrobial mechanism of microemulsions were studied and were found to be involved in the growth inhibition curve, relative conductivity, protein, and nucleic acid leakage.

#### 3.6.1. Growth Curve Analysis

**Figure 5:**
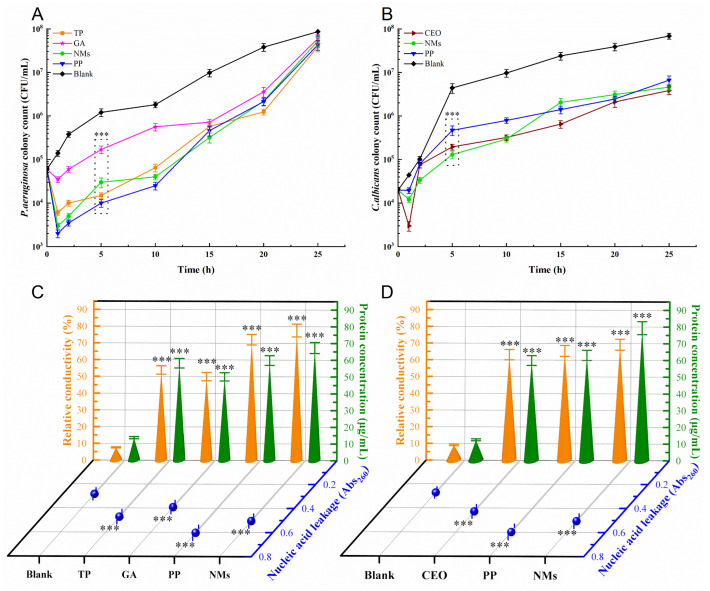
Bacteriostatic mechanism data of NMs. (**A**) Growth curve of *P. aeruginosa*, (**B**) growth curve of *C. albicans*, (**C**) cell membrane permeability of *P. aeruginosa*, (**D**) cell membrane permeability of *C. albicans*. PP was selected as a positive control, and no bacteriostatic agent was selected as a blank control. Each value represents means ± SD (*n* = 3). *** indicates statistical difference at *p* < 0.001 level compared with blank group.

The growth inhibition curve could explore the effect of preservatives on the bacterial or fungal growth cycle to gain a preliminary understanding of their inhibitory effect. The antimicrobial activity of the microemulsion was assessed by counting viable microbes in suspensions of *P. aeruginosa* and *C. albicans* at various time intervals after contact with the samples. The effects of NMs on the growth of *P. aeruginosa* and *C. albicans* are shown in Figure 5A,B. Compared with the blank control, NMs and the antimicrobial active ingredients have a specific inhibitory effect on *P. aeruginosa* and *C. albicans*. Compared with the positive control, the antimicrobial effect of NMs was basically the same as that of PP. This result was consistent with the MIC values shown in Table 1. Specifically, TP, PP, and NMs had the same MIC value against *P. aeruginosa*. The characteristic reflected in the growth curve was that their growth curves had the same trend (Figure 5A). The MIC value of NMs against *C. albicans* was smaller than that of PP. The characteristic reflected in the growth curve was that the fungistatic effect of NMs was better than that of PP (Figure 5B). Neither deionized water nor Tween 80 (1% *v/v*) exhibited any inhibitory effect, which indicated that it was TP, GA, and CEO (rather than dispersal medium) that had antimicrobial activity. However, the growth curve of *C. albicans* showed that the fungistatic activity of NMs was not as good as that of CEO after 10 h. This may be influenced by surfactants (Tween 80) and co-surfactants (glycerol). Ma et al. showed that Tween 80 and soybean oil reduced the antibacterial activity of cinnamon bark oil [44,47]. Furthermore, all treatments with antimicrobial ingredients had initial reductions in the counts of *P. aeruginosa* and *C. albicans*, whereas NM-treated *P. aeruginosa* and *C. albicans* took longer to recover to initial colony counts. It may be that the microemulsion and antimicrobial ingredients have killed some of the original bacteria or fungi at the tested concentrations and then affected the cellular structure or biochemical reactions of the growing bacteria or fungi. Once the bacteria and fungi overcome the inhibition, they will multiply rapidly [45]. The above results showed that NMs may have stable broad-spectrum antimicrobial activity; its inhibitory effect was comparable to that of PP, and could potentially replace PP.

#### 3.6.2. Cell Membrane Permeability Analysis

The growth curve macroscopically reflected the growth-inhibitory effect of the microemulsion on *P. aeruginosa* and *C. albicans*, while cell membrane permeability experiments may reveal the microemulsion’s antimicrobial mechanism from the microscopic level. The extracellular relative conductivities of *P. aeruginosa* and *C. albicans* treated with different samples are shown in Figure 5C,D. The relative conductivity of the blank control group did not change much, all within 10%. This may be due to the normal lysis and death of bacteria and fungi, resulting in an increase in the relative conductivity [35]. Compared with the blank control, the relative conductivities of *P. aeruginosa* and *C. albicans* bacterial suspensions changed significantly after adding samples. It is intended that the addition of antimicrobial active ingredients will increase the permeability of the cell membrane, resulting in the loss of electrolytes, including K^+^, Ca^2+^, Na^+^, and so on [35]. Compared with the positive control, the relative conductivities of the NM-treated suspensions of *P. aeruginosa* and *C. albicans* were basically at the same level as PP. It was shown that the effect of NMs on the cell membrane was comparable to that of PP. This result is consistent with the trend reflected in the above growth curves.

The standard curve of bovine serum albumin was y = 0.0101x + 0.0244 (R^2^ = 0.9998), and the curve had a good linear relationship, which was used to measure the protein content in the bacterial or fungal suspension. The results of the extracellular protein concentration of *P. aeruginosa* and *C. albicans* treated with different samples are shown in Figure 5C,D. The protein content of the blank control group was relatively low, all within 15 μg/mL. This may be due to the normal lysis of cells and transmembrane transport of proteins. Compared to the blank control, the protein content of *P. aeruginosa* and *C. albicans* suspensions changed significantly after adding samples. It is intended that the addition of bacteriostatic or fungistatic active ingredients disrupts the integrity of the cell membrane, resulting in a massive loss of intracellular proteins. Compared with the positive control, the protein content of NM-treated suspensions of *P. aeruginosa* and *C. albicans* was slightly higher than that of PP. The results showed that NMs had slightly more damaging effects on cell membranes than PP. This result was basically consistent with the trend reflected by the relative conductivity. In addition, the protein content of NM-treated *P. aeruginosa* suspension was significantly higher than that of TP and GA; the protein content of NM-treated *C. albicans* suspension was also higher than that of CEO.

The results of extracellular nucleic acid leakage of *P. aeruginosa* and *C. albicans* treated with different samples are shown in Figure 5C,D. The results of Abs260 were basically consistent with the results of relative conductivity and protein content. Notably, the Abs260 values of NM-treated *P. aeruginosa* and *C. albicans* suspensions were slightly lower than those of parabens. This result did not correspond to the trend reflected by relative conductivity and protein content. This was because the maximum absorption peak of PP was also near 260 nm, which interfered with the determination of the amount of nucleic acid leakage.

**Figure 6:**
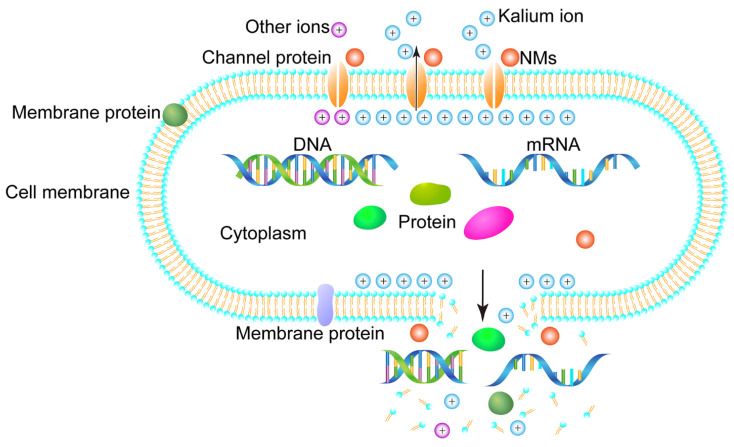
Schematic diagram of the antimicrobial activity mechanism of NMs.

The research results on the antimicrobial mechanism of microemulsion in this paper shown that the compounded microemulsion achieves the inhibitory effect by changing the permeability of the cell membrane and destroying the integrity of the membrane. The schematic diagram of the antimicrobial activity mechanism of NMs is shown in Figure 6.

The interaction between NMs and the cell membrane changed the permeability and integrity of the cell membrane, leading to the loss of intracellular ions, proteins, nucleic acids, and other substances and accelerating the cell death. The growth curve reflected the growth-inhibition effect of the microemulsion on bacteria and fungi from a macroscopic perspective. The relative conductivity, protein leakage, and nucleic acid leakage reflected the destructive impact of the microemulsion on bacterial and fungal cell membranes from a microscopic perspective. Moreover, relative conductivity, leakage of proteins, and nucleic acids have all confirmed that the antimicrobial effect of the microemulsion was more advantageous than that of a single antimicrobial component. Razdan et al. reported that levofloxacin could enhance the antibacterial activity of clove oil nano-emulsions and the stronger disrupting effect of the nano-emulsion on the biofilm of *P. aeruginosa* [48]. Moreover, Almasi et al. demonstrated that a microemulsion of thyme essential oil and propionic or acetic acid may have synergistic effects [49]. This showed that it is effective to formulate the antimicrobial active ingredients into a microemulsion with broad-spectrum antimicrobial activity. The dramatic changes in relative conductivity, protein, and nucleic acid leakage were the result of the interaction of the microemulsion with cell membrane components. Nano-scale microemulsions have a large surface area, which facilitates the dissolution of cell membranes and penetrates better than antibacterial active ingredients, thereby disrupting the integrity of cell membranes [50,51]. The results of this study were consistent with the antibacterial mechanism reported in the literature previously. The interaction of bacteriostatic substances with bacterial cell membranes will increase membrane permeability. The change in bacterial membrane permeability was always be accompanied by the loss of intracellular substances, especially the loss of some ions and 260-nm-absorbing materials [52,53]. The leakage of intracellular ions led to changes in the relative conductivity of extracellular solutions, which indirectly reflected changes in cell membrane permeability. Diao et al. studied the mechanism of inhibition of fennel seed essential oil against *S. dysenteriae*, describing it as the essential oil first breaking through the permeability of the cell membrane, which is associated with the disruption of integrity of the generalized membrane, resulting in leakage of electrolytes as well as proteins, reducing sugars and nucleic acids [35]. In addition, Bajpai et al. also showed that essential oils exert their inhibitory effects through cell membrane permeability associated with a general membrane-disrupting effect, mainly manifested as a reduction in the number of viable bacteria, loss of absorbing materials at 260 nm, and leakage of potassium ions [54]. Although the antimicrobial mechanism of essential oil microemulsion has been reported in this paper and other literature, some issues still need to be revealed in follow-up scientific research. For example, studying the bacteriostatic or fungistatic components of essential oils and the proteins leaked by bacteria or fungi is necessary.

## 4. Conclusions

This study used TP, GA, and CEO as antibacterial active ingredients. NMs with broad-spectrum antimicrobial activity, high antioxidant activity, high stability, and non-irritant qualities were successfully constructed. The optimal ratio of CEO, glycerin, Tween 80, and water in NMs was 13.5%:13.5%:50%:23%, in which the concentration of TP was 50 mg/mL and GA was 100 mg/mL. The antimicrobial test results showed that NMs had significant inhibitory capacity against *S. aureus*, *P. aeruginosa*, *E. coli*, *C. albicans*, and *A. brasiliensis*, with MIC values of 156 μg/mL, 62.5 μg/mL, 125 μg/mL, 250 μg/mL, and 125 μg/mL, respectively. The bacteriostatic and fungistatic effect were basically equivalent to that of PP. Antioxidative test results showed that NMs had superior antioxidative properties against DPPH free radicals comparable to those of vitamin E and the microemulsion was more effective than a single component. NMs still showed good antimicrobial activity after 90 days of storage at different temperatures. Except for *S. aureus*, the inhibitory zone decreased within 20%.

Furthermore, the irritation of NMs was lower than that of PP, which is relatively safe. It was proven that NMs might achieve their antimicrobial effect by changing the permeability of the cell membrane and destroying the integrity of the cell membrane. Moreover, the cell membrane permeability test confirmed a synergistic effect between the active components of the microemulsion, which provides a theoretical basis for the compounding of natural antimicrobial components in microemulsions or nano-emulsions. Since NMs are composed of multiple active ingredients, there may be more than one mechanism of antimicrobial action. Further work is required to fully understand the mechanisms involved to demonstrate the viability of NMs as natural antimicrobial agents in food, drugs, and cosmetics.

## Figures and Tables

**Table 1:** The minimum inhibitory concentration value of each active antimicrobial ingredient.

Sample	MIC (μg/mL)				
	*S. aureus*	*E. coli*	*P. aeruginosa*	*C. albicans*	*A. brasiliensis*
TP	15.60	62.50	125.00	-	-
GA	31.25	2.50	15.60	-	-
CEO	200	390	100	100	630
PP	125.00	125.00	125.00	500.00	31.25
NMs	156.00	62.50	125.00	250.00	125.00

Note: “-” indicates that the minimum inhibitory concentration of the substance could not be detected within the experimental concentration range.

## Data Availability

Data is contained within the article.

## Supplementary Materials

The following supporting information can be downloaded at: https://www.mdpi.com/article/10.3390/microorganisms11010002/s1, Figure S1: The effect of different concentrations of TP on the NMs; Figure S2: The effect of different concentrations of GA on the NMs; Figure S3. Diagram of the effect of different ratios of co-surfactant phase and oil phase on NMs; Table S1: The effect of different concentrations of TP on the antibacterial effect of NMs (the highest content of CEO); Table S2: The effect of different concentrations of TP on the antibacterial effect of NMs (the content of CEO is 1.5%); Table S3: Influence of the ratio of CEO to glycerin phase on the antibacterial effect of NMs (the highest content of CEO).
