# Supplementary material for: Microemulsion of Cinnamon Essential Oil Formulated with Tea Polyphenols, Gallic Acid, and Tween 80: Antimicrobial Properties, Stability and Mechanism of Action
Source: Microorganisms. 2022 Dec 20;11(1):2. doi: 10.3390/microorganisms11010002 (PMC9867123; doi:10.3390/microorganisms11010002)
Supplement: Supplementary file 1 [file microorganisms-11-00002-s001.zip › microorganisms-2012156-supplementary.pdf]

microorganisms

Supplemental Data

2. Materials and Methods

2.2. Establishment of NMs

2.2.1. Determination of TP Content

The ratio of CEO to glycerin was fixed at 1:3 as the oil phase. Pure water was used as blank control, and TP solutions of different concentrations were selected as the water phase. The oil phase and Tween-80 were mixed in 9 beakers in a mass ratio of 1:9, 2:8, 3:7, 4:6, 5:5, 6:4, 7:3, 8:2, and 9:1. NMs were determined when the formulation turned into a limpid liquid with low viscosity after being gradually diluted with water. Record the water phase volume and draw a pseudo-ternary phase diagram.

2.2.2. Determination of GA Content

Determine the concentration of TP according to the results in 2.2.1. Glycerol was used as a blank control; the CEO was mixed with GA solutions of different concentrations in a ratio of 1:3 as the oil phase. The oil phase and Tween-80 were mixed in 9 beakers in a mass ratio of 1:9, 2:8, 3:7, 4:6, 5:5, 6:4, 7:3, 8:2, and 9:1. When the formulation became transparent and viscous with low viscosity, the formulation was determined to be a microemulsion. Record the water phase volume and draw a quasi-ternary phase diagram.

2.2.3. Determination of Cosurfactant Phase to Oil Phase Ratio

Based on the results in 2.2.1 and 2.2.2, TP and GA concentrations were determined. The CEO was mixed with glycerin in the ratios of 1:1, 1:2, and 1:3 as the oil phase. The oil phase and Tween-80 were mixed in 9 beakers in a mass ratio of 1:9, 2:8, 3:7, 4:6, 5:5, 6:4, 7:3, 8:2, and 9:1. Once the mixture reached a transparent liquid with a low viscosity, water was gradually added until microemulsions were formed. Record the water phase volume and draw a quasi-ternary phase diagram.

3. Results and Discussion

3.2. Analysis of Antimicrobial Properties of NMs

3.2.1. The effect of TP Content

The effect of the added amount of TP in the water phase on the NMs is shown in Figure S1, and pure water is used as a blank control. The blue area is the microemulsion area. Using the analysis function in Origin 2022, the absolute areas of Figure S1. are calculated as 0.1406, 0.1316, 0.1038, and 0.0777, respectively and the absolute area of the blank control is 0.1669. In this NMs, the size of the microemulsion phase range decreases with the increase of the TP aqueous solution; that is, the greater the amount of TP added in the water phase, the more unstable the NMs is. During the experiment, it was found that the addition of Tween-80 would cause the precipitation of TP in water, possibly because the water solubility of Tween-80 reduced the solubility of TP in water.

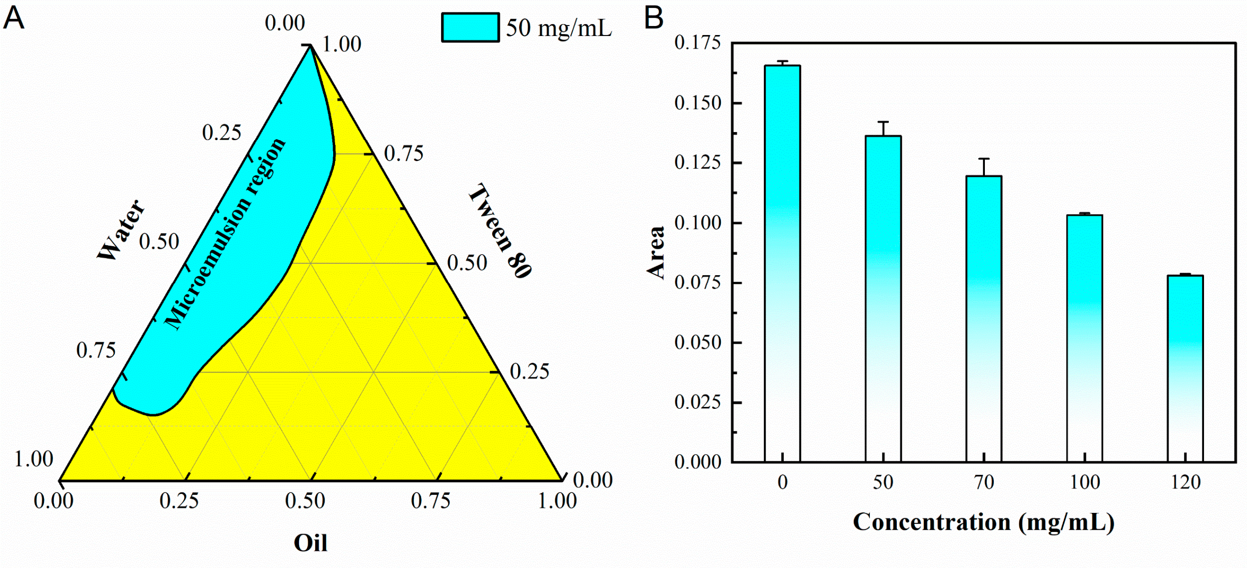

Figure S1. The effect of different concentrations of TP on the NMs. (A) Quasi-ternary phase diagram with TP concentration of 50 mg/mL, (B) Relationship between microemulsion phase area and TP concentration.

Table S1. The effect of different concentrations of TP on the antimicrobial effect of NMs (the highest content of CEO).

Concentration (mg/mL)	Inhibition Zone Diameter (mm)				
	S. aureus	*E. coli*	*P. aeruginosa*	*C. albicans*	*A. brasiliensis*
0	17	13	12	50	35
50	30	40+	28	64	48
70	20	38	14	56	38
100	20	32	15	54	32
120	24	30	15	52	35

Note: "+" indicates that the inhibition zone of the substance is greater than the experimental range.

Table S2. The effect of different concentrations of TP on the antimicrobial effect of NMs (the content of CEO is 1.5%).

Concentration (mg/mL)	Inhibition Zone Diameter (mm)				
	S. aureus	*E. coli*	*P. aeruginosa*	*C. albicans*	*A. brasiliensis*
0	16	13	12	50	35
50	17	35	13	50	38
70	20	35	14	52	32
100	20	37	18	54	32
120	25	40+	22	50	35

Note: "+" indicates that the inhibition zone of the substance is greater than the experimental range.

Table S1 shows the results of the NMs antimicrobial experiment with the highest proportion of CEO in the microemulsion phase area. The concentrations of TP are 0, 50, 70, 100, and 120 mg/mL, and the corresponding highest proportions of CEO are 2.3%, 4.3%, 3.5%, 2.5%, and 1.5%. Table S2 shows the results of the NMs antimicrobial experiment with CEO accounting for 1.5% in the microemulsion phase area. Table S1 shows that

the diameter of the inhibition zone of *S. aureus*, *E. coli*, *P. aeruginosa*, *C. albicans*, and *A. brasiliensis* obviously increased with increased CEO. Table S2 shows that in the NMs with the same proportion of CEO, the diameter of the inhibition zone of each strain is positively correlated with the concentration of TP in the water phase. Considering the above results, the final selection of the consistency of TP in the water phase is 50 mg/mL.

3.2.2. The Effect of GA Content

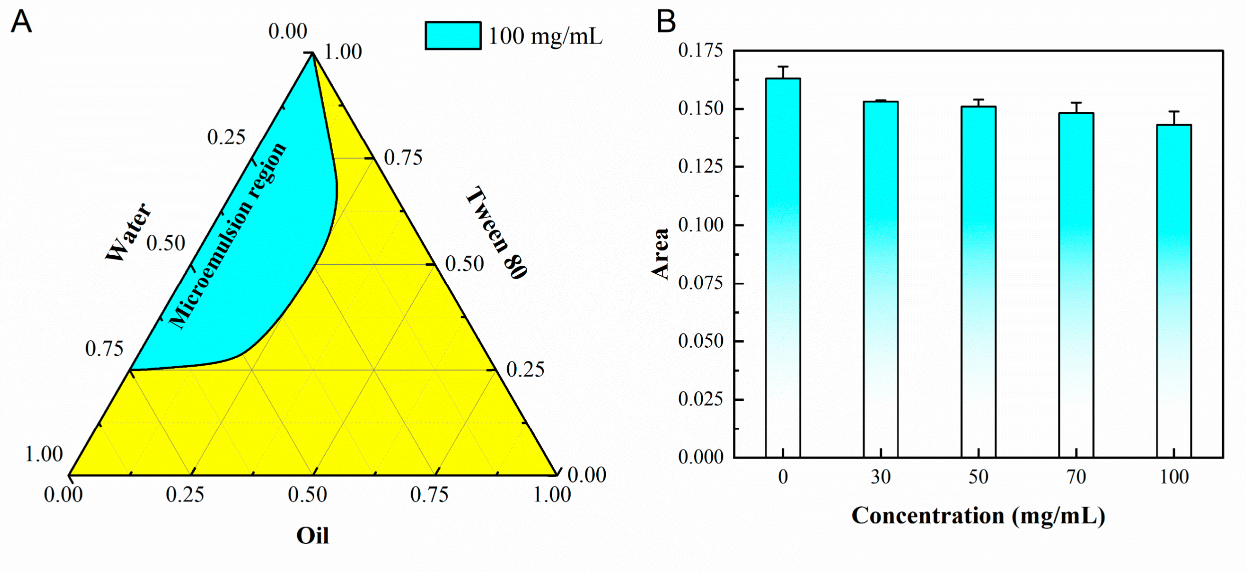

Figure S2. The effect of different concentrations of GA on the NMs. (A) Quasi-ternary phase diagram with GA concentration of 100 mg/mL, (B) Relationship between microemulsion phase area and GA concentration.

TP solution (50mg/mL) was used as the water phase, Tween-80 was used as a surfactant, and a mixture of CEO and glycerol (1:3) was used as the oil phase. The concentration gradient of GA was set to 30 mg/mL, 50 mg/mL, 70 mg/mL and 100 mg/mL based on the solubility of GA in glycerol of 100 mg/mL. The effect of different concentrations of GA on the NMs is shown in Figure S2. The absolute area of the microemulsion phase area was calculated as 0.1595, 0.1535, 0.1531, 0.1514, and 0.1507. Compared with the blank control group, the area of the microemulsion phase was not much different. To ensure that the NMs have high antimicrobial activity, the final concentration of GA in glycerol was 100 mg/mL.

3.2.3. Influence of the Ratio of Essential Oil and Cosurfactant on NMs

As for the TP solution (50 mg/mL) in the water phase, the concentration of GA in glycerol is 100 mg/mL, Tween-80 is the emulsifier, and the mixture of CEO and glycerin is the oil phase. The effect of the ratio of CEO to co-emulsifier on NMs is shown in Figure S3. Its area is 0.1477, 0.1440, 0.1507. It can be concluded that the change in glycerol content has a certain influence on NMs. When the weight ratio of CEO and glycerin is 1:3, the area of microemulsion phase is the largest. It can be seen that although the polyol has no surface activity, it has a certain solubilizing effect on the NMs. Table S3 shows the antimicrobial test results of the NMs with the highest proportion of CEO in the microemulsion phase area. In terms of inhibiting *S. aureus*, *E. coli*, and *P. aeruginosa*, the antimicrobial effect of CEO and glycerol in the 1:1 and 1:2 has little difference, and the antimicrobial effect of 1:3 is relatively better. It may be related to the proportion of CEO. To ensure the antimicrobial effect of CEO, the content of CEO should be increased as much as possible; that is, the ratio of CEO and glycerin phase should be 1:1.

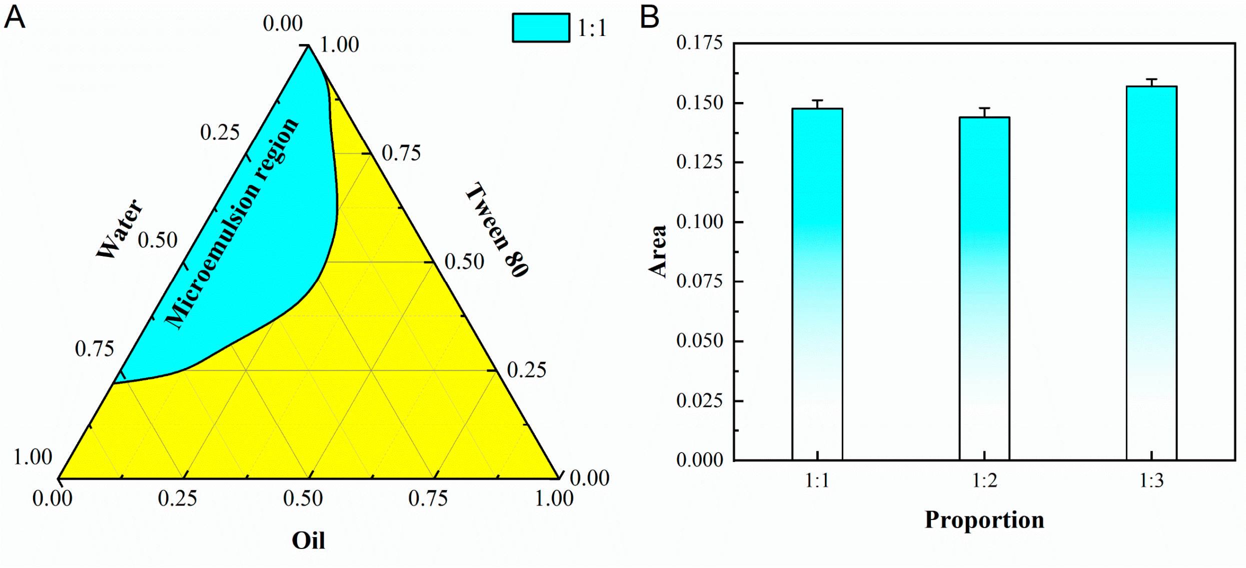

Figure S3. Diagram of the effect of different ratios of co-surfactant phase and oil phase on NMs. (A) Quasi-ternary phase diagram with the 1:1 ratio of co-surfactant phase to oil, (B) The relationship between the area of the microemulsion phase and the ratio of co-surfactant to oil.

Table S3. Influence of the ratio of CEO to glycerin phase on the antimicrobial effect of NMs (the highest content of CEO).

Ratio	Inhibition Zone Diameter (mm)				
	S. aureus	*E. coli*	*P. aeruginosa*	*C. albicans*	*A. brasiliensis*
1:1	39	48	37	65+	60+
1:2	42	46	38	64	48
1:3	31	38	36	65+	50

Note: "+" indicates that the inhibition zone of the substance is greater than the experimental range.

Combined with all the experimental results, it was finally determined that the proportions of CEO, glycerol phase, Tween-80, and water phase in the NMs of the natural extract complex microemulsion system constructed in this paper were 13.5%, 13.5%, 50%, and 23%. The concentration of GA in the glycerol phase is 100 mg/mL, and the concentration of TP in the aqueous phase is 50 mg/mL.
